# A Narrative Review of the Herbal Preparation of Ayurvedic, Traditional Chinese, and Kampō Medicines Applied as Radioprotectors

**DOI:** 10.3390/antiox12071437

**Published:** 2023-07-17

**Authors:** Blanca Ibáñez, Ana Melero, Alegría Montoro, Juan F. Merino-Torres, Jose M. Soriano, Nadia San Onofre

**Affiliations:** 1 Food & Health Laboratory, Institute of Materials Science, University of Valencia, 46980 Paterna, Spain; blancaibanezdepedro@gmail.com (B.I.); sanonofre.nadia@gmail.com (N.S.O.); 2 Department of Pharmacy and Pharmaceutical Technology and Parasitology, Faculty of Pharmacy, University of Valencia, 46100 Burjassot, Spain; ana.melero@uv.es; 3 Service of Radiological Protection, Clinical Area of Medical Image, University and Polytechnic La Fe Hospital, 46026 Valencia, Spain; almonpas@hotmail.com; 4 Biomedical Imaging Research Group GIBI230, Health Research Institute (IISLaFe), University and Polytechnic La Fe Hospital, 46026 Valencia, Spain; 5 Joint Research Unit on Endocrinology, Nutrition and Clinical Dietetics, Health Research Institute La Fe, University of Valencia, 46026 Valencia, Spain; merino_jfr@gva.es; 6 Department of Endocrinology and Nutrition, University and Polytechnic La Fe Hospital, 46026 Valencia, Spain; 7 Department of Medicine, Faculty of Medicine, University of Valencia, 46010 Valencia, Spain; 8 Department of Community Nursing, Preventive Medicine and Public Health and History of Science, University of Alicante, 03690 Alicante, Spain

**Keywords:** herbal preparations, radioprotection, Ayurveda, traditional Chinese medicine, Kampō medicines

## Abstract

In recent years, there has been growing scientific interest in the search for natural radioprotectors that can be used to mitigate the effects of radiation on patients, healthcare personnel, and even for space travel. This narrative review covers the past fifty years and focuses on herbal preparations of Ayurvedic, Traditional Chinese, and Kampō Medicines that have the potential to reduce or eliminate the harmful effects of radiation. Our findings highlight ten herbal preparations, namely Abana, Amalakyadi Churna, Amritaprasham, Brahma, Bu-zhong-yi-qi-tang (BZYQT), Chyavanaprasha, Cystone, Geriforte, Mentat, and Triphala, which have demonstrated potential radioprotective effects. This review examines their composition, properties, and possible mechanisms of action in relation to their radioprotective properties. Exploring the ethnobotany of traditional Asian medicine is particularly interesting as it may lead to the discovery of new active compounds with radioprotective properties.

## 1. Introduction

In the past 30 years, there has been a significant shift in attention towards evaluating plant products as radioprotectors [1,2]. This change is primarily due to their effectiveness in providing protection against radiation, coupled with their low toxicity [3]. Plant extracts are believed to possess radioprotective properties because they contain numerous active constituents, such as antioxidants, immunostimulants, and compounds with antimicrobial activity [4]. Consequently, the screening of herbal drugs has become a major focus in the search for new drug discoveries [5]. When exploring the use of natural radioprotectors, it is essential not only to study the active compounds or natural organisms individually but also to investigate preparations or combinations of compounds derived from natural sources [6]. In recent years, the number of publications on these natural preparations has grown exponentially, with literature based on Ayurvedic and Traditional Chinese (or Kampō) medicines readily available. This availability allows for the exploration of possible synergistic effects among the plants included in the preparation and their active components [7]. The aim of this study is to conduct a narrative review of traditional preparations from Ayurvedic, Traditional Chinese, and Kampō (defined as the Japanese study and adaptation of traditional Chinese medicine) Medicines, focusing on their composition, properties, and possible mechanisms of action.

## 2. Materials and Methods

In this study, we conducted an electronic search of PubMed, Scopus, and Web of Science for literature published over the last 50 years, updated to 1 June 2023. A combination of the following keywords was used “herbal preparations” OR “Ayurveda” OR “Traditional Chinese” OR “Kampō” AND “Radioprotection” OR “Radioprotector” OR “Radiation”. We further conducted a screening process by examining the full-text articles of the shortlisted abstracts to determine eligibility. Articles for which the full text was not accessible or not available in English were excluded. To ensure accuracy and consistency, two pairs of reviewers (B.I., A. Melero., N.S.O., J.M.S.), who have expertise in medical and health assessment as well as training in research methodology, independently screened three sections: titles, abstracts, and full texts (if eligible). The reviewers evaluated the generalizability of the studies and collected data from the selected articles. Any disagreements that arose were resolved by another pair of reviewers (J.-F.M.-T., A. Montoro). Additionally, several subsections were created in the results and discussion section to define each herbal formulation with their respective botanical names.

## 3. Results and Discussion

### 3.1. Abana

Abana is an Ayurvedic formulation containing *Terminala arjuna*, *Nepeta hindostana*, *Withania somnifera*, *Commiphora mukul*, *Centella asiatica*, *Phyllanthus emblica*, *Terminalia chebula*, and *Glycyrrhiza glabra* [8]. In humans, this herbal preparation reduces hypertension [9,10] and other cardiovascular diseases [11], as well as inhibiting platelet aggregation [12]. Its ability as a scavenger of free radicals, specifically nitric oxide [13], has been demonstrated, and it is used in the prevention of neurodegenerative diseases such as Alzheimer’s [14] and Parkinson’s disease [15]. Sasikumar and Devi [16] conducted a study on the induction of myocardial infarcts in rats, demonstrating that Abana had an effect on modulating lipid peroxidation and increasing the antioxidant and detoxifying systems. This drug, which induces infarcts in rats, was found to have a protective effect on mouse bone marrow against the formation of micronuclei induced by radiation [17]. The alcoholic extract of Abana (20 mg/kg bw) provides protection against gastrointestinal damage and increases survival in mice after exposure to γ-radiation. Acute toxicity studies revealed that Abana exhibited no toxicity at a dose of 1.6 g/kg body weight, and no mortality was observed following the administration of the drug. The LD_50,_ in mice, of the Abana was 1.8 g/kg bw. This study demonstrated the ability of Abana as a radioprotective agent, with the optimal radioprotective dose 1/90 according to the LD_50_ in mice [8,18].

### 3.2. Amalakyadi Churna

The popular treatise, called Sharangdhar Samhita, formulation contains *Phyllanthus emblica*, *Plumbago zeylanica*, *Terminalia chebula*, *Piper longum*, and rock salt (halite) [19]. The therapeutic indication of Amalakyadi Churna is applied to anorexia, dyspepsia, fever, and indigestion [20]. Trigar et al. [21] conducted a study on the antitumor efficacy of *T. chebula* ethanolic extract using the Ehrlich Ascites Carcinoma tumor model. They observed a significant increase in body weight, survival time, and lifespan, along with a reduction in the incidence rate of tumors at early stages. In a study by Reddy [22] involving Swiss albino mice, the LD_50_ of the extract was found to be 313 mg/kg bw i.p. when administered intraperitoneally. However, subacute toxicity was not observed when the mice were treated with 25 mg/kg bw i.p. of the extract for 30 days. It is noteworthy that *T. chebula* is the sole plant in this herbal preparation that exhibited radioprotective activity [23]. Naik et al. [24] observed that the aqueous extract of this herbal mixture inhibited the formation of γ-radiation-induced strand breaks in plasmid DNA pBR322. In fact, this study analyzed possible radioprotective compounds in this extract, with ascorbate, gallic acid, and ellagic acid being detected. These phytochemicals inhibited xanthine/xanthine oxidase activity and scavenged of 2,2,1-diphenyl-1-picrylhydrazyl (DPPH) radicals. Gandhi and Nayar [25] discovered the free radical neutralizing ability of the extract, which was demonstrated by its protective effect on plasmid DNA pBR322. The extract prevented the conversion of the supercoiled form of the plasmid to the open circular form, a change that occurs as a result of radiation damage. Furthermore, when administered at a dose of 80 mg/kg bw i.p., the extract exhibited the ability to minimize oxidative-stress-induced lipid peroxidation in the liver membranes of mice. It also decreased radiation-induced damage to DNA, both in vivo and in vitro, when exposed to 2 Gy of γ-radiation [26].

### 3.3. Amritaprasham

Amritaprasham is an Ayurvedic herbal preparation containing *Asparagus recemosus*, *Boerhaavia diffusa*, *Cinnamomum zeylanica*, *Clerodendrum serratum*, *Elettaria cardamomum*, *Embelica officinalis*, *Garcinia morella*, *Glycyrrhiza glabra*, *Hedychium spicatium*, *Holstemma annulare*, *Macuna pruriens*, *Mesua ferrea*, *Phaseolus adenanthus*, *Phylanthus niruri*, *Piper longum*, *P. nigrum*, *Purerira tuberose*, *Saccharum officinalum*, *Sida retusa*, *Vigna vexilata*, *Vitis vinifera*, and *Zingiber officinale* [27] applied to improve stamina and strength, retard aging, provide hemopoietic stimulatory action, and reduce symptoms such as anorexia, bronchial asthma, burning sensation, cough, epistaxis, erectile dysfunction, fever, gastrointestinal disorders, hemorrhoids, loss of consciousness, menstrual disorders, seminal abnormalities (including azoospermia and oligospermia), thirst, urinary disorders, and vomiting [28]. Vayalil et al. [29] conducted preclinical studies on the administration of Amritaprasham and observed positive effects on mice exposed to radiation. The treatment resulted in a reduction in radiation-induced weight loss and prevented a decrease in the weight of important organs such as the liver, kidney, and spleen. Furthermore, the administration of Amritaprasham showed significant effects on biochemical markers. On the second day after irradiation, treatment with Amritaprasham resulted in a significant decrease of 36% in serum glutamate pyruvate transaminase (GPT), a marker of liver function. Additionally, there was a reduction of 57% in serum lipid peroxide levels and a decrease of 40% in hepatic lipid peroxide levels. By the seventh day after irradiation, treatment with Amritaprasham continued to show positive effects. The levels of serum GPT were reduced by 55%, while serum lipid peroxide levels decreased by 40% and hepatic lipid peroxide levels decreased by 60%. These findings suggest that Amritaprasham may have a protective effect against radiation-induced damage, as indicated by the improvement in organ weights and the reduction in biochemical markers associated with oxidative stress and liver function. 

### 3.4. Brahma

Brahma has several fruits, such as *E. officinalis* and *T. chebula*, and it has been applied as a specific geriatric drug that targets the brain, with regular consumption believed to offer several cognitive benefits [30]. It is thought to enhance mental clarity, increase resilience to mentally demanding tasks, and improve memory and cognition. Additionally, it is believed to have antiaging effects, potentially reducing the symptoms associated with aging, such as wrinkles and graying of hair [28]. In animal studies, this product has demonstrated protective effects against radiotoxicity, reducing the loss of organ weight (kidney, liver, and spleen) and body weight. It also decreased levels of serum and liver lipid peroxides, alkaline phosphatase, and GPT [28]. Vayalil et al. [29] observed that the oral administration of Brahma increased levels of lymphocytes and neutrophils in cancer patients undergoing radiotherapy. Furthermore, it was associated with a decrease in leukopenia, neutropenia, lymphopenia, and serum lipid peroxidation levels.

### 3.5. Bu-Zhong-Yi-Qi-Tang (BZYQT)

Bu-zhong-yi-qi-tang (BZYQT) is a formulation that differs in its composition if we use traditional Chinese medicine or Kampō medicine (defined as the Japanese study and adaptation of traditional Chinese medicine) [31]. For the first case, it is composed of the following plants: *Angelica sinensis*, *Astragalus membranaceus*, *Atractylodes macrocephala*, *Bupleurum chinense*, *Cimicifuga foetida*, *Citrus reticulata*, *Glycyrrhiza uralensis*, *Panax ginseng*, *Zingiber officinale*, and *Ziziphus ziziphus* [32]. In the case of Kampō medicine, it contains a mixture of *Angelica acutiloba*, *A. membranaceus*, *Atractylodes lancea*, *Bupleurum falcatum*, *Cimicifuga simplex*, *C. reticulata*, *G. uralensis*, *P. ginseng*, *Z. officinale*, and *Z. zizyphus* [33]. BZYQT is utilized for the treatment of various chronic diseases. It has been found to be effective in managing conditions such as chronic fatigue [34], respiratory tract injury [35], male infertility [36], and respiratory allergy [37]. Additionally, BZYQT is useful in treating gastrointestinal ailments such as gastrectasia and chronic diarrhea [38]. Studies have demonstrated its ability to improve digestive system function [39] and restore vitality in rats with spleen-qi deficiency [40]. Moreover, BZYQT has shown a protective effect on intestinal mucosa in mice with 5-FU-induced intestinal mucositis. Its molecular mechanism involves inhibiting cytokine-mediated apoptosis or necrosis, thereby reducing the gastrointestinal side effects of cancer chemotherapy [39]. Therefore, BZYQT holds promise as an alternative treatment for patients experiencing gastrointestinal side effects from cancer chemotherapy. Furthermore, the oral administration of BZYQT has been shown to regulate the function of immune cells [41] and suppress serum IgE levels in animal models of allergy [42]. The antitumor activity of BZYQT was demonstrated, by Ito and Shimura [43], in mice with Ehrlich-Lettre ascites carcinoma, and they observed that it suppressed the growth of these carcinomas when administered intraperitoneally, prolonging the survival of mice with this type of carcinoma. The anticancer activity was studied [44] in mouse females, where BZYQT showed an inhibitory effect on the carcinogenesis of the mouse endometrium. Two short (0.2% daily feed for two weeks) and long-term (divided into four groups: with different diets with and without the formulation) experiments were performed. In both experiments, there was a decrease in the expression levels of membrane receptors, such as c-jun, tumor necrosis factor-K (TNF-K), and estrogen receptors. 

The bibliography indicates that BZYQT exhibits antiproliferative activity specifically against human hepatoma cell lines, while not affecting healthy human hepatocytes [45]. It achieves this by arresting the cell cycle in G0/G1 phases and inhibiting DNA synthesis, leading to apoptosis in hepatoma cells, but it does not have the same effect on healthy human hepatocytes [45]. These authors, one year before [46], detected the immunomodulatory effect of BZYQT, in vitro, and demonstrated that it is capable of stimulating the granulocyte growth factor and colonizer and the tumor necrosis factor-α in peripheral blood mononuclear cells in healthy volunteers and in patients with hepatocellular carcinoma, since they can modulate their cytokines.

BZYQT has been found to have a protective effect against radiation-induced damage. When mice were exposed to different doses of radiation, the symptoms varied in terms of onset time, severity, and maximum duration depending on the radiation dose. In mice irradiated at high (12 Gy) and intermediate (6.5 Gy) doses, the protective effect of BZYQT on the intestine and bone marrow was investigated. This was done by conducting a survival test on the jejunum crypts to assess the protection of the intestine, and by measuring the formation of colonies in the endogenous spleen to evaluate the protection of the bone marrow. In mice irradiated at low doses (2 Gy), they examined the effect on apoptosis of intestinal crypt cells [47] and concluded that the administration of BZYQT before irradiation protected the crypts, increased the formation of colonies in the spleen, and reduced the induction of apoptosis.

### 3.6. Chyavanaprasha

Chyavanaprasha is a renowned Ayurvedic polyherbal preparation that consists of more than 50 plants [48]. Its name is derived from its inventor, sage Chyavana, and its historical evidence can be found in an Ayurvedic text called Charak Samhita. It is widely known as the ‘elixir of life’ and is highly regarded for its numerous health benefits. Chyavanaprasha is considered a valuable formulation for enhancing overall health and vitality in individuals of all age groups. It is commonly used to address issues such as cough, dyspnea, voice problems, and cardiac ailments, among others [49]. Chyavanaprasha has been found to possess radioprotective effects in studies. The administration of graded doses of hydroalcoholic extract of Chyavanaprasha, ranging from 5 to 80 mg/kg bw, for 5 consecutive days prior to exposure to a high dose of γ-radiation (10 Gy) protected animals from radiation-induced sickness and mortality [50]. The most effective dose observed was 15 mg/kg, where the highest survival rate found was 58% on day 30 after irradiation. Importantly, the Chyavanaprasha extract showed no signs of toxicity at doses up to 6 g/kg bw, indicating its safety and lack of systemic toxicity [50]. Furthermore, studies have demonstrated other potential benefits of Chyavanaprasha, such as to reduce liver damage induced by carbon tetrachloride in rats [51]. In addition, Chyavanaprasha has been found to decrease ascites (abnormal fluid buildup) and solid tumor volume in animals with tumors. This effect leads to an increase in their lifespan [52]. In a separate study, bidi smokers, who consumed 20 g of Chyavanaprasha twice a day for two months, experienced a decrease in coughing, an increase in appetite, and weight gain [53]. These findings indicated that Chyavanaprasha not only exhibits radioprotective properties but also demonstrates potential hepatoprotective effects and potential benefits in conditions related to cancer.

### 3.7. Cystone

Cystone is an herbal formulation of *Achyranthes aspera, Cyperus scariosus*, *Didymocarpus pedicellata*, *Rubia cordifolia*, *Saxifraga ligulate*, and *Tinospora cordifolia* [54]. It is very effective in maintaining the proper function of the urinary tract. It reduces susceptibility to urinary problems by preserving the integrity of mucous membranes. Additionally, Cystone helps to maintain optimal liver and urinary tract irrigation, thereby supporting the optimal performance of these organs. Cystone inhibits lithogenesis by reducing the formation of stones produced by substances such as oxalic acid, causing their expulsion by micro-spraying [55]. The roots of *R. cordifolia* have astringent, antibacterial, and anti-inflammatory activity; this latter activity is also observed in *A. aspera*. The oil from the roots of *C. scariosus* has shown anti-inflammatory properties [56,57]. Cystone has a high antioxidant power, so much so that it is used as a reference standard in the evaluation of the antioxidant activity of other plants [58]. In fact, Rao et al. [59] suggested its use with cisplatin due to that Cystone help, in laboratory mice, to decrease blood urea nitrogen and serum creatinine after five days of treatment with cisplatin without disturbing cisplatin function at a specific dose. Several years after in a randomized clinical trial on patients with cancer, El-Ghiaty et al. [60] studied that treatment combined of Cystone plus cisplatin produced significantly decreased levels of blood urea nitrogen, serum creatinine, serum cystatin C, and nephrotoxic side effects compared to treatment with cisplatin alone. The radioprotective activity of Cystone has been investigated in vivo. In a study, mice were treated with different doses of Cystone for five consecutive days prior to irradiation. The treatment resulted in a delay in the onset of mortality and a reduction in the symptoms associated with irradiation syndrome [61].

### 3.8. Geriforte

Geriforte is a formulation based on the following plants: *Withania somnifera*, *Phyllanthus emblica*, and *Mucura urens*. It was originally described for its antistress properties [62], and, subsequently, in laboratory animals, it was observed that it had antitumor and antiviral effects [63,64]. In the clinical trial conducted by Banerjee et al. [65], they evaluated the efficacy and safety of Geriforte, determining its antioxidant efficacy in geriatric patients evaluating a series of enzymatic activities such as superoxide dismutase, catalase, glutathione peroxidase, glutathione reductase, reduced glutathione, and malondialdehyde. The authors concluded that they found no adverse effects and that it was effective as an antioxidant. In another clinical trial [66], Geriforte also showed its antioxidant action by protecting the erythrocyte membrane of healthy patients from free radicals. Geriforte supplementation resulted in an increase in catalase enzyme activity in erythrocytes and reduced cytotoxicity caused by free radicals. However, in healthy patients, Geriforte-based supplements did not alter the activity of erythrocyte superoxide dismutase or plasma levels of antioxidant agents. These findings suggest that Geriforte possesses cytoprotective properties in erythrocytes, primarily attributed to its specific antioxidant activity, which primarily involves protecting the cell membrane’s protection systems and intracellular communications. Jagetia et al. [67] studied, in vitro, several herbal formulations, Geriforte being the one with the most powerful antioxidant capacity, to sequester free radicals of nitrogen. Bansal et al. [68] showed a reduction in cytotoxicity and apoptosis, in addition to an inhibition of lipid peroxidation and capacity to maintain high levels of endogenous antioxidants, suggesting that this antioxidant activity could be due to its cytoprotective and immunomodulatory properties. Pathania et al. [69] found an increase in the activity of several enzymes (catalase, superoxide dismutase, and glutathione peroxidase) in the liver of mice fed for one month with Geriforte (1 g for 4 weeks). The treatment of mice with different concentrations of Geriforte, 5 consecutive days before irradiation, delays the onset of mortality and reduces the symptoms of irradiation syndrome. A dose of 10 mg/kg bw protects against gastrointestinal syndrome and death or the depletion of bone marrow cells with 1.14 of the dose reduction coefficient for γ-radiation [70].

### 3.9. Mentat

The Mentat, also called BR-16A, is a formulation based on the following plants: *Adoxa moschatellina*, *Bacopa monnieri*, *Centella asiatica*, *Mucuna urens*, *Phyllanthus emblica*, *Terminalia arjuna*, and *Withania somnifera* [71]. Mentat has been used to regulate behavior, improve memory, and minimize the loss associated with aging [72,73]. Geriforte has been found to maintain brain function in normal situations and under emotional and mental pressures [74,75]. It improves mental functions by modulating cholinergic and GABAergic neurotransmission, which is associated with improvements in mental quotient, memory span, concentration ability, and stress threshold. It also provides benefits in treating insomnia and correcting speech defects. [76]. It acts as a neuroprotective agent due to antioxidant and free-radical-scavenging properties; therefore, it could be used for the rehabilitation of patients that have suffered from an ischemic stroke [77]. Demir et al. [78] observed that propolis could prevent cataractogenesis in ionizing radiation-induced cataracts in the lenses of rats in comparison with caffeic acid phenethyl ester, *Nigella sativa* oil and thymoquinone.

The radioprotective action of this herbal formulation was demonstrated by Jagetia and Baliga [79], where they administered several doses of an ethanolic extract of Mentat, five consecutive days prior to the irradiation of mice, and showed that it reduces their mortality as well as the disease caused by the exposure to radiation, protecting even against gastrointestinal syndrome, observing maximum protection against this syndrome at a concentration of 80 mg/kg bw. In addition, the evaluation of acute toxicity showed that Mentat was not toxic at a dose of 1.5 g/kg bw. The LD_50_ dose of Mentat was 1.75 g/kg in mice. This radioprotective action is associated synergically with its antioxidant activity due to its capacity to sequester these type of free radicals, as well as products of these, inhibiting the pathological conditions generated by the generation of these radicals [71].

### 3.10. Triphala

It is a combination of the preparation of three tropical fruits comprised of equal parts of formulation based on three plants: *Phyllanthus emblica*, *Terminalia bellerica*, and *T. chebula* [80,81]. It is a formulation very rich in antioxidants, and with antibacterial, antimalarial, antifungal, antiallergic, antiviral, and antitumor properties [82]. The antioxidant activity of Triphala has been studied in an aqueous extract of the formula, finding a powerful inhibition of lipid peroxidation and an important potential to scavenger, in vitro, hydroxyl and superoxide radicals [83]. Kumar et al. [84] attribute its high antioxidant activity to its phenolic content (gallic acid and tannins identified as major components). Subsequently, Mahesh et al. [85] evaluated the preventive effects of aqueous extracts of *T. chebula* on the oxidative status in the liver and kidney of elderly rats compared to young rats. The concentrations of malondialdehyde, lipofuscin, carbonyloprotein, vitamins C and E, and the activities of xanthine oxidase, manganese superoxide dismutase, catalase, glutathione peroxidase, glutathione reductase, glutathione-S-transferase, and glucose-6-phosphate dehydrogenase were used as biomarkers. The administration of the aqueous extract of *T. chebula* modulated the oxidative stress and antioxidant status in the liver and kidney of elderly rats. Sandhya et al. [86] studied, in vitro, the antitumor activity of Triphala and observed that it inhibits the growth of tumor cells. The exogenous addition of antioxidants (glutathione and N-acetyl-cysteine) caused the inhibition of the antiproliferative capacity of Triphala in the tumor lines due to the fact that it induces reactive oxygen species and induces apoptosis in the lines studied. These same authors [87,88] demonstrated, in vivo, that the direct oral administration of Triphala to mice (40 mg/kg bw) transplanted with thymic lymphomas produces a significant reduction in tumor growth, taking as a marker of the effect the tumor volume. It was also discovered that apoptosis was significantly higher in the tumor tissue, suggesting its involvement in the reduction in tumor growth. This antitumor activity has been studied in more detail, particularly with one of the components of Triphala, *P. emblica*. Its aqueous extract was cytotoxic for tumor cells by interaction with cell cycle regulation. Both Triphala and *T. chebula* are not genotoxic using two DNA damage tests, VITOTOX and the comet assay [89]. In the VITOTOX test, none of the extracts were identified as genotoxic. In the comet assay, the extracts of *T. chebula* and Tripahala significantly increased DNA damage at a concentration greater than 500 μg/mL. This is not considered contradictory because the DNA damage in the comet assay may not be permanent and therefore does not have to lead to mutations. Kaur et al. [73] evaluated its cytotoxicity in an acetone extract of Triphala, demonstrating its cytotoxic potential on several cancer cell lines, attributing this effect to gallic acid. Russell et al. [90] showed that phenolic constituents (including gallic acid) are three times more cytotoxic than Triphala for tumor cells, while cytotoxicity in normal cells was low.

Triphala has been found to increase the survival rate of mice and alleviate the symptoms associated with irradiation syndrome following exposure to γ-radiation. Additionally, it offers protection against death caused by gastrointestinal and hematopoietic syndrome [91]. Triphala protects mice against lethality induced by radiation by sequestering, dose-dependent, free radical action. The effect of 10 mg/kg of Triphala extract was studied in mice exposed to γ-ray doses between 7 and 12 Gy (1 time a day, 5 consecutive days before irradiation), for which reduced mortality and disease symptoms due to radiation occurred. It has a 1.15 dose reduction factor for γ rays [92]. Sandhya et al. [86] observed that radiation-induced mortality was reduced by 60% in mice fed Triphala (1 g/kg bw/day) orally for 7 days before the body’s total exposure to 7.5 Gy, in addition to continuing with this dose for a week post-irradiation. Yoon et al. [93] published the radioprotective effect of Triphala against damage to the intestinal mucosa in rats by exposure to ionizing radiation being administered Triphala 1 g/kg bw/day orally for 5 consecutive days in a group of rats before irradiation, and another group was given Triphala 1 and 1.5 g/kg/day orally for 10 consecutive days. The damage to the rectal mucosa was induced by a single dose of 12.5 Gy of γ-irradiation on the fifth day. All rats were sacrificed at 10 days, and histological changes in the surface epithelium were evaluated; it was shown that high doses (1.5 g/kg bw/day) of Triphala improved the damage induced by the radiation and had a significantly higher degree of recovery at the level of histological changes.

## 4. Conclusions

In our study of the scientific literature focused on ten herbal preparations, including Abana, Amalakyadi Churna, Amritaprasham, Brahma, Bu-zhong-yi-qi-tang (BZYQT), Chyavanaprasha, Cystone, Geriforte, Mentat, and Triphala, we found that they have radioprotective properties which could be useful to patients or healthcare personnel exposed to radiation, and even astronauts for future space travel. Table 1 and Table 2 reflected the ingredients of herbal preparations and their (human, animal, or dose) studies, respectively. The discovery of herbal preparations with radioprotective properties from Ayurvedic, Traditional Chinese, and Kampō Medicines opens up avenues for further exploration in Asian and other regions’ traditional treatments using plants for radiation protection. It is crucial to explain different herbal preparations, as has been suggested by several authors [94,95], and to elucidate their mechanisms of action to facilitate both basic and applied research on natural radioprotectors based on herbal preparations. This research will not only verify their efficacy in radiation protection but also shed light on other potential effects of their usage.

## Figures and Tables

**Table 1:** Herbal preparation from Ayurvedic, Traditional Chinese, and Kampō Medicines applied as radioprotectors and their ingredients.

Products	Ingredients
Abana	*Terminala arjuna*, *Nepeta hindostana*, *Withania somnifera*, *Commiphora mukul*, *Centella asiatica*, *Phyllanthus emblica*, *Terminalia chebula*, and *Glycyrrhiza glabra.*
Amalakyadi Churna	*Phyllanthus emblica*, *Plumbago zeylanica*, *Terminalia chebula*, *Piper longum*, and rock salt (halite).
Amritaprasham	*Asparagus recemosus*, *Boerhaavia diffusa*, *Cinnamomum zeylanica*, *Clerodendrum serratum*, *Elettaria cardamomum*, *Embelica officinalis*, *Garcinia morella*, *Glycyrrhiza glabra*, *Hedychium spicatium*, *Holstemma annulare*, *Macuna pruriens*, *Mesua ferrea*, *Phaseolus adenanthus*, *Phylanthus niruri*, *Piper longum*, *P. nigrum*, *Purerira tuberose*, *Saccharum officinalum*, *Sida retusa*, *Vigna vexilata*, *Vitis vinifera*, and *Zingiber officinale.*
Brahma	*Embelica officinalis* and *Terminalia chebula.*
Bu-zhong-yi-qi-tang (BZYQT)	*In the case of traditional Chinese medicine including Angelica sinensis*, *Astragalus membranaceus*, *Atractylodes macrocephala*, *Bupleurum chinense*, *Cimicifuga foetida*, *Citrus reticulata*, *Glycyrrhiza uralensis*, *Panax ginseng*, *Zingiber officinale*, and *Ziziphus ziziphus*. In the case of Kampō medicine incluidng *Angelica acutiloba*, *A. membranaceus*, *Atractylodes lancea*, *Bupleurum falcatum*, *Cimicifuga simplex*, *C. reticulata*, *G. uralensis*, *P. ginseng*, *Z. officinale*, and *Z. ziziphus.*
Chyavanaprasha	Polyherbal preparation that consists of more than 50 plants.
Cystone	*Achyranthes aspera*, *Cyperus scariosus*, *Didymocarpus pedicellata*, *Rubia cordifolia*, *Saxifraga ligulate*, and *Tinospora cordifolia.*
Geriforte	*Withania somnifera*, *Phyllanthus emblica*, and *Mucura urens.*
Mentat	*Adoxa moschatellina*, *Bacopa monnieri*, *Centella asiatica*, *Mucuna urens*, *Phyllanthus emblica*, *Terminalia arjuna*, and *Withania somnifera.*
Triphala	*Phyllanthus emblica*, *Terminalia bellerica*, and *T. chebula.*

**Table 2:** References selected from each studied herbal preparations, according to components, human, animal, and dose studies.

Products	Components	Human Studies	Animal Studies	Dose Studies
Abana	[8]	[9,10,11,12,13,14,15]	[16,17,18]	[8,18]
Amalakyadi Churna	[19]	[20,23]	[21,25,26]	[22,24]
Amritaprasham	[27]	[28,29]	[29]	[28]
Brahma	[30]	[28,30]	[28,29]	
Bu-zhong-yi-qi-tang (BZYQT)	[31]	[33,34,35,36,37,38,45,46]	[32,39,40,42,43,44,47]	[41]
Chyavanaprasha	[48]	[49,53]	[50,51,52]	
Cystone	[54]	[55,56,57,60,61]	[58,59]	
Geriforte		[65,66]	[63,64,68,69]	[70,71,92,93]
Mentat	[72]	[73,74,78]	[73,75,79]	[80,91]
Triphala	[81,82,83]	[87,88,90]	[85]	[80,88,89,91]

## Abbreviations

bw	Body weight
BZYQT	Bu-zhong-yi-qi-tang
DNA	Deoxyribonucleic acid
DPPH	2,2,1-diphenyl-1-picrylhydrazyl
GPT	Glutamate pyruvate transaminase
Gy	Gray
i.p.	Intraperitoneal
IgE	Immunoglobulin E
LD50	Lethal dose 50
TNF-K	Tumor necrosis factor-K
